# Integration of primary care and hospital care

**Published:** 2012-09-04

**Authors:** Bert Groot Roessink, Jeltje Schraverus

**Affiliations:** Board of Directors and CEO Zorggroep Almere, The Netherlands; Chairwoman of the Executive Board of the Hospital Flevoziekenhuis in Almere, The Netherlands

**Keywords:** integrated health, long-term care, structural integration, integration primary care and hospital care

## Abstract

See the powerpoint presentation for the abstract.

Powerpoint presentation available at http://www.integratedcare.org at congresses – San Marino – programme.

